# Bmp7 Maintains Undifferentiated Kidney Progenitor Population and Determines Nephron Numbers at Birth

**DOI:** 10.1371/journal.pone.0073554

**Published:** 2013-08-26

**Authors:** Mayumi Tomita, Misako Asada, Nariaki Asada, Jin Nakamura, Akiko Oguchi, Atsuko Y. Higashi, Shuichiro Endo, Elizabeth Robertson, Takeshi Kimura, Toru Kita, Aris N. Economides, Jordan Kreidberg, Motoko Yanagita

**Affiliations:** 1 Department of Nephrology, Graduate School of Medicine, Kyoto University, Kyoto-city, Kyoto, Japan; 2 Department of Pharmacology, Kansai Medical University, Moriguchi-city, Osaka, Japan; 3 Sir William Dunn School of Pathology, University of Oxford, Oxford, United Kingdom; 4 Department of Cardiovascular Medicine, Graduate School of Medicine, Kyoto University, Kyoto-city, Kyoto, Japan; 5 Kobe City Medical Center General Hospital, Kobe-city, Hyogo, Japan; 6 Regeneron Pharmaceuticals, Inc., Tarrytown, New York, United States of America; 7 Children’s Hospital Boston, Harvard Medical School, Boston, Massachusettes, United States of America; The University of Manchester, United Kingdom

## Abstract

The number of nephrons, the functional units of the kidney, varies among individuals. A low nephron number at birth is associated with a risk of hypertension and the progression of renal insufficiency. The molecular mechanisms determining nephron number during embryogenesis have not yet been clarified. Germline knockout of *bone morphogenetic protein 7 (Bmp7)* results in massive apoptosis of the kidney progenitor cells and defects in early stages of nephrogenesis. This phenotype has precluded analysis of Bmp7 function in the later stage of nephrogenesis. In this study, utilization of conditional null allele of *Bmp7* in combination with systemic inducible *Cre* deleter mice enabled us to analyze Bmp7 function at desired time points during kidney development, and to discover the novel function of Bmp7 to inhibit the precocious differentiation of the progenitor cells to nephron. Systemic knockout of *Bmp7 in vivo* after the initiation of kidney development results in the precocious differentiation of the kidney progenitor cells to nephron, in addition to the prominent apoptosis of progenitor cells. We also confirmed that *in vitro* knockout of *Bmp7* in kidney explant culture results in the accelerated differentiation of progenitor population. Finally we utilized colony-forming assays and demonstrated that Bmp7 inhibits epithelialization and differentiation of the kidney progenitor cells. These results indicate that the function of Bmp7 to inhibit the precocious differentiation of the progenitor cells together with its anti-apoptotic effect on progenitor cells coordinately maintains renal progenitor pool in undifferentiated status, and determines the nephron number at birth.

## Introduction

Over the last decade, the impacts of prenatal conditions on health and disease in later life have been subjects of intense research [1]. Low birth weight is associated with hypertension and a higher risk of diabetes, cardiovascular diseases, and chronic kidney disease [2]. Animal studies and human epidemiological data support the hypothesis that low birth weight is associated with a congenital deficit in nephron number, which results in compensatory glomerular hypertrophy and an increased susceptibility to renal disease progression [3], [4], [5]. The molecular mechanisms determining the number of nephrons, however, remain largely unknown.

The embryonic kidney is derived from the intermediate mesoderm and formed by a reciprocal induction between the cap mesenchyme and ureteric bud [6]. Cap mesenchyme condenses around a tip of the ureteric bud, undergoes epithelialization to form renal vesicles, and differentiates to form most parts of the nephron: podocytes, proximal tubules, Henle’s loops, and distal tubules. Simultaneously, the ureteric bud extends and branches to form collecting ducts. Molecular analysis and genetic studies have identified the presence of renal progenitor cells in the cap mesenchyme surrounding the tips of the branching ureteric buds [4], [7], [8], [9], [10].

Bone morphogenetic protein 7 (Bmp7) is a morphogen expressed in the kidney. Germline *Bmp7* knockout mice exhibited aplastic kidneys with a few nephrons, leading to perinatal death [11], [12]. Because nephrogenesis was arrested at an early stage in these mice, the precise function of Bmp7 during kidney development remained undetermined.

The expression domains of *Bmp7* in the developing kidney were analyzed utilizing *Bmp7^+/LacZ^* mice [11], [13]. LacZ staining revealed that *Bmp7* is widely expressed in cap mesenchyme, ureteric buds, and some derivatives of cap mesenchyme, podocytes and distal tubules. Given that various types of cells in the developing kidney express *Bmp7*, coupled with the fact that Bmp7 is a secreted factor, we surmised that the deletion of *Bmp7* in any specific cell type might not result in a prominent phenotype. We therefore opted for ubiquitous global ablation of *Bmp7* at desired time points as a strategy to define its role in kidney maturation.

In our study, the utilization of the conditional null allele of *Bmp7* in combination with systemic inducible *Cre* deleter mice enabled us to analyze Bmp7 function at different time points during kidney development. While the knockout of *Bmp7* before the initiation of kidney development recapitulates germline *Bmp7* knockout mice (**Figure S1 in the supplementary material**), the knockout of *Bmp7* after the initiation of kidney development allowed us to demonstrate the novel phenotype, precocious differentiation of progenitor cells. We also confirmed that Bmp7 inhibits differentiation of renal progenitor cells utilizing kidney explant culture and colony-forming assay. Taken together, we demonstrated for the first time that Bmp7 maintains nephron progenitor cells in the undifferentiated state, thereby influencing the number of nephrons at birth.

## Results

### Acceleration of nephron maturation and apoptosis of cap mesenchyme at E14.5 in Bmp7 knockout kidneys

To analyze the role of Bmp7 in kidney development, we utilized *Bmp7* conditional knockout mice [14] and systemic inducible *Cre* (*Gt(ROSA)26Sor^CreERT2^*) mice. We bred *Bmp7^+/LacZ^*;*Gt(ROSA)26Sor^CreERT2^* mice with *Bmp7^fl/fl^* mice to yield *Bmp7^LacZ/fl^*;*Gt(ROSA)26Sor^CreERT2^* (*Bmp7* knockout) and *Bmp7^+/fl^*;*Gt(ROSA)26Sor^CreERT2^* (control) embryos. We knocked down *Bmp7* expression through the administration of tamoxifen to pregnant mothers bearing both types of embryos. Because of the toxicity of the systemic expression of *CreERT2* on hematological tissues in *Gt(ROSA)26Sor^CreERT2^* mice [15], all experiments were designed such that both the *Bmp7* knockout and control embryos carry *CreERT2*.

**Figure 1:**
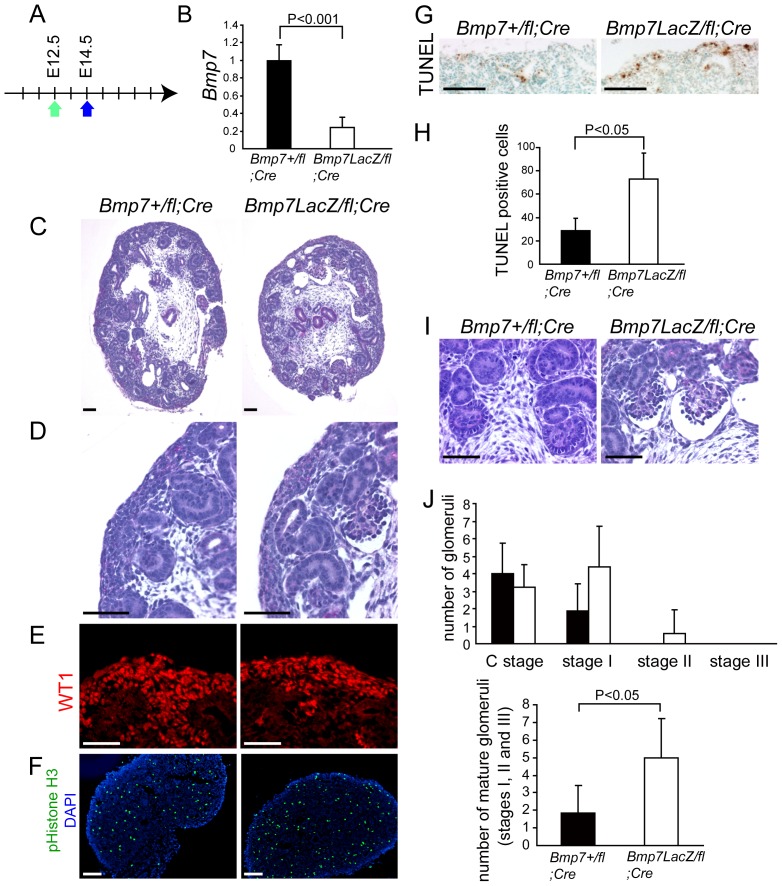
Acceleration of nephron maturation and apoptosis of cap mesenchyme at E14.5 in Bmp7 knockout kidneys. (**A**) Pregnant mothers bearing Bmp7^LacZ/fl^;Gt(ROSA)26Sor^CreERT2^ and Bmp7^+/fl^;Gt(ROSA)26Sor^CreERT2^ embryos were administered tamoxifen at E12.5, and sacrificed at E14.5. (**B**) The expression of Bmp7 mRNA was significantly reduced at E14.5 in Bmp7^LacZ/fl^;Gt(ROSA)26Sor^CreERT2^ (Bmp7 knockout kidneys) (n = 4, white column) compared to control kindeys (n = 5, black column). Data are represented as mean ± SD. (**C and D**) Cap mesenchyme was maintained in both Bmp7 knockout kidneys and control kidneys. (**E**) Cap mesenchyme positive for WT1 was maintained in both genotypes. (**F**) The distribution and the number of phospho-Histone H3-positive cells were comparable in both genotypes. pHistone H3 denotes phospho-Histone H3. (**G**) TUNEL-positive cells were increased in Bmp7 knockout kidneys. (**H**) The number of TUNEL-positive mesenchymal cells per section was increased in knockout kidneys (white column) compared to control kidneys (black column). Data are represented as mean ± SD. The average numbers of TUNEL-positive mesenchymal cells in three sections were used as the values for each embryo. The mean of the values from three embryos is presented in the graph. (**I**) Bmp7 knockout kidneys exhibited inappropriately “mature” glomeruli at E14.5. (**J**) Glomeruli in the mature stages were increased in Bmp7 knockout kidneys (white column) compared to the control kidneys (black column). Criteria for the assessment of glomerular maturity are detailed in method section. Because C stage is the most common in control kidneys at E14.5, we summed up the glomeruli of stages I, II, and III for comparison of maturation. Glomeruli were counted in 5 slices of each kidney. The mean of the values ± SD is presented in the graphs (n = 7 for control embryos, and 5 for knockout embryos). Scale bars: 100 µm (**C, F, G**) or 50 µm (**D, E, I**). n.s.: not significant.

We administered tamoxifen to pregnant mice at embryonic day 12.5 (E12.5), a time when the epithelialization of cap mesenchyme has begun, and analyzed the embryos at E14.5 (**Figure 1A**). The knockout efficiency of *Bmp7* in the kidney was approximately 80% (**Figure 1B**). At this time point, the sizes of the kidneys as well as the volume of cap mesenchyme of both genotypes were comparable (**Figure 1C-E and S** **2**). While the proliferation of metanephric mesenchyme was similar between the two genotypes (**Figure 1F**), the apoptosis of metanephric mesenchyme was clearly increased in *Bmp7* knockout kidneys (**Figure 1G, H**).

At E14.5, very few “mature” glomeruli were observed in control kidneys, whereas the number of “mature” glomeruli was increased in knockout kidneys (**Figure 1I**). We carefully analyzed the numbers of glomeruli in each stage of maturity with the serial sections of the kidney at 100 µm intervals according to the method previously described [16], [17]. Glomeruli in the mature stages (stages I, II, and III) significantly increased in knockout kidneys **(** **Figure 1J** **)**.

### Acceleration of nephron maturation and reduction of cap mesenchyme at E18.5 in Bmp7 knockout kidneys

**Figure 2:**
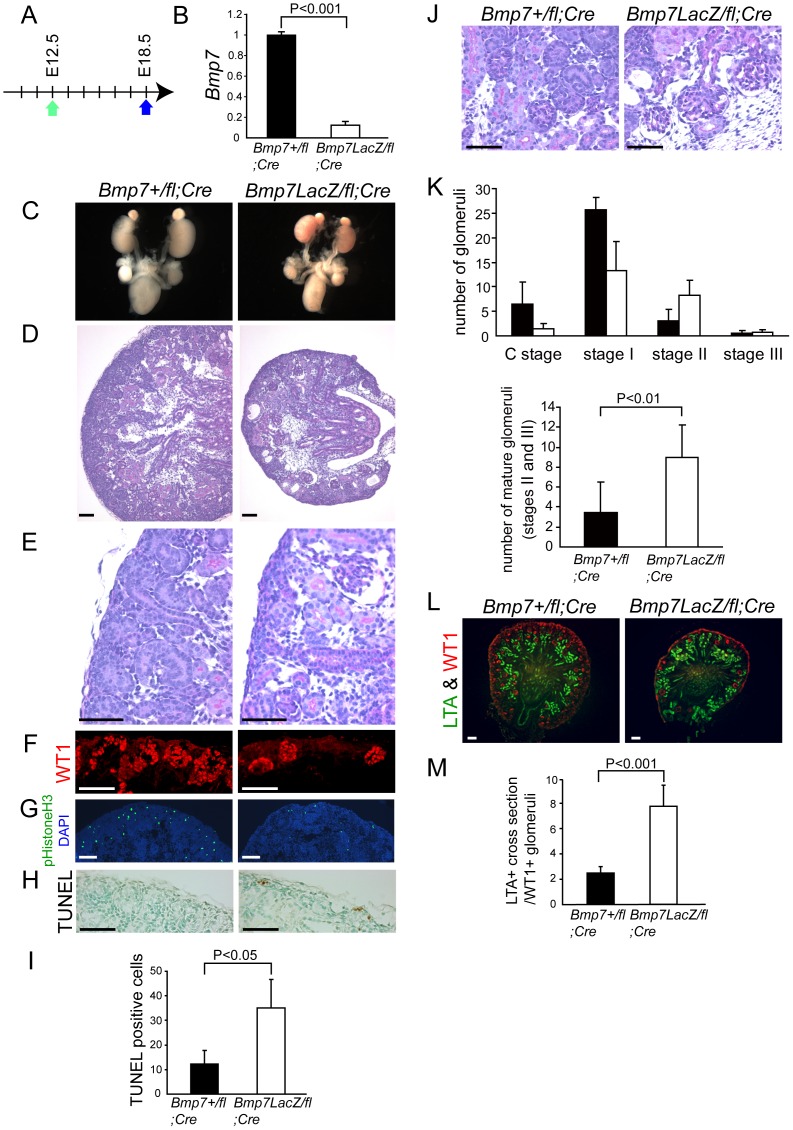
Acceleration of nephron maturation and reduction of cap mesenchyme at E18.5 in Bmp7 knockout kidneys. (**A**) Pregnant mothers bearing Bmp7^LacZ/fl^;Gt(ROSA)26Sor^CreERT2^ and Bmp7^+/fl^;Gt(ROSA)26Sor^CreERT2^ embryos were administered tamoxifen at E12.5, and sacrificed at E18.5. (**B**) The expression of Bmp7 mRNA was significantly reduced in knockout kidneys (n = 4, white column) compared to control kidneys (n = 3, black column). Data are represented as mean ± SD. (**C and D**) Bmp7 knockout embryos at E18.5 exhibited small kidneys. (**E and F**) The number of cap mesenchymal cells, as shown by the immunostaining of WT1, was decreased in Bmp7 knockout kidneys. (**G**) The number of phospho-Histone H3-positive cells was reduced in Bmp7 knockout kidneys. pHistone H3 denotes phospho-Histone H3. (**H**) TUNEL-positive mesenchymal cells were increased in Bmp7 knockout kidneys. (**I**) The number of TUNEL-positive mesenchymal cells per section was increased in knockout kidneys (white column) compared to control kidneys (black column). Data are represented as mean ± SD. The average numbers of TUNEL-positive mesenchymal cells in three sections were used as the values for each embryo. The mean of the values from three embryos is presented in the graph. (**J**) Inappropriately “mature” glomeruli were observed in Bmp7 knockout kidneys. (**K**) Glomeruli in the mature stages were increased in Bmp7 knockout kidneys (white column) compared to the control kidneys (black column). Criteria for the assessment of glomerular maturity are detailed in method section. Because stage I is the most common in control kidneys at E18.5, we summed up the glomeruli of stages II and III for comparison of maturation. Glomeruli were counted in 3 slices of each kidney. The mean of the values ± SD is presented in the graphs (n = 4 for control embryos, and 6 for knockout embryos). (**L**) Kidneys were stained with LTA (green) and WT1 (red) to label proximal tubules and glomeruli, respectively. The volume of LTA-positive proximal tubule sections in knockout kidneys was comparable to the control kidneys, whereas the number of glomeruli was significantly reduced. (**M**) The number of LTA^+^ proximal tubule cross sections normalized by the number of WT1^+^ glomeruli was increased in knockout kidneys (white column) compared to control kidneys (black column). Data are represented as mean ± SD. At least three sections were stained for each kidney. The sum of the number of LTA-positive proximal tubule cross sections was divided by the sum of the number of WT1-positive glomeruli. The mean of the values from four embryos is presented in the graph. Scale bars: 100 µm (**D, G, L**) or 50 µm (**E, F, H, J**).

Next we analyzed the embryos at E18.5, six days after the administration of tamoxifen (**Figure 2A**). The knockout efficiency of *Bmp7* in the kidney was approximately 90% (**Figure 2B**). *Bmp7* knockout embryos had smaller kidneys with fewer nephrons than control embryos (**Figure 2C, D, and S** **2**). Histological analysis revealed a significant reduction in the volume of cap mesenchyme in knockout kidneys (**Figure 2E and S** **2**), which was also confirmed by immunostaining of WT1, a marker for cap mesenchyme (and podocytes) (**Figure 2F and S** **2**). The proliferation of knockout kidneys appeared to be decreased (**Figure 2G**), which is likely due to the depletion of cap mesenchyme. The apoptosis of metanephric mesenchyme increased in knockout kidneys (**Figure 2H, I**). Sustained apoptosis of metanephric mesenchyme from E14.5 in knockout kidneys likely accounts for the depletion of cap mesenchyme at E18.5.

Maturation of glomeruli in knockout kidneys observed at E14.5 was more prominent at E18.5. Inappropriately “mature” glomeruli were observed in knockout kidneys at E18.5 (**Figure 2J**), and glomeruli in mature stages (stages II and III) significantly increased in knockout kidneys (**Figure 2K**).

During the maturation of nephron, proximal tubules elongate and become convoluted in the cortex. Kazama *et al*. previously utilized the number of proximal tubular cross-sections divided by the number of glomeruli as an index of proximal tubule complexity and maturation [18]. We analyzed the index in both genotypes, and found that the complexity of proximal tubules was higher in knockout kidneys (**Figure 2L, M**). We also found that the number of distal tubular cross-sections divided by the number of glomeruli tended to increase in knockout kidneys, although the difference was not statistically significant **(Figure S3)**. These results excluded the possibility that the reduction of Bmp7 changes the proportion of resulting nephron by increasing the proximal tubules and decreasing the distal tubules. Taken together, maturation of the nephrons was accelerated in knockout kidneys.

### Bmp7 inhibits the differentiation of cap mesenchyme in kidney explant culture

**Figure 3:**
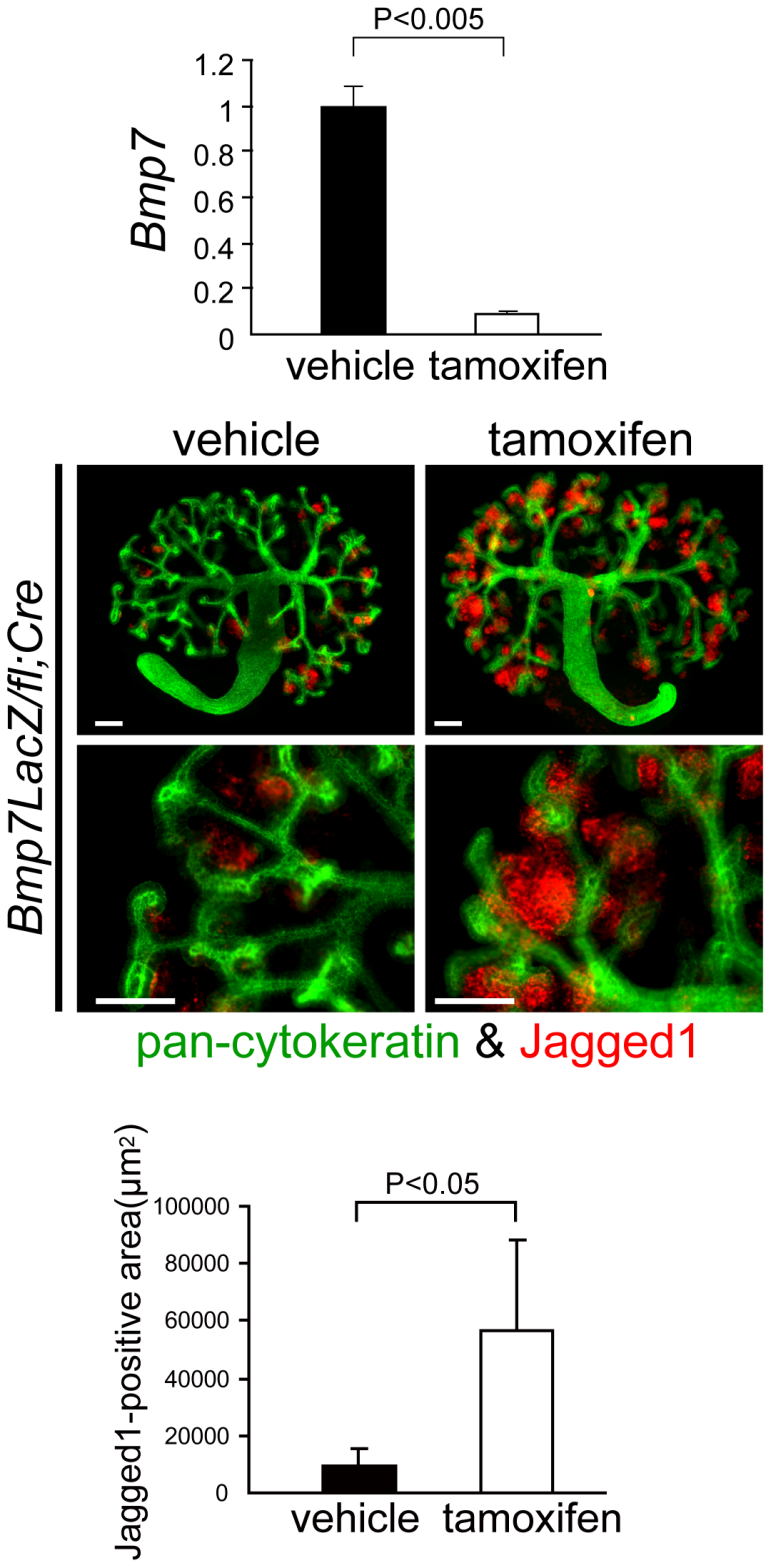
Bmp7 inhibits the differentiation of cap mesenchyme in kidney explant culture. Kidney explants were taken from Bmp7^LacZ/fl^;Gt(ROSA)26Sor^CreERT2^ embryos at E12.5 and cultured for 72 h in the presence or absence of 4-OHT. The expression of Bmp7 mRNA was significantly reduced in Bmp7^LacZ/fl^;Gt(ROSA)26Sor^CreERT2^ explants treated with 4-OHT compared to the explants from the same embryos treated with a vehicle (n = 3). Data are represented as mean ± SD. Whole explants were costained with pan-cytokeratin (green) to label ureteric buds and Jagged1 (red) to label developing nephrons. The Jagged1-positive red area was measured utilizing Photoshop software. In Bmp7^LacZ/fl^;Gt(ROSA)26Sor^CreERT2^ explants treated with 4-OHT, Jagged1-positive regions were significantly expanded, indicating accelerated differentiation. Scale bars: 100 µm.

The epithelialization and subsequent differentiation of cap mensenchyme is the first step of nephrogenesis. We hypothesized that the precocious maturation of nephron segments in *Bmp7* knockout kidneys is due to the accelerated differentiation of cap mesenchyme. To test this hypothesis, we employed kidney explant cultures, a well-characterized culture system utilized to analyze kidney development *ex vivo*. Kidney explants were harvested from *Bmp7^LacZ/fl^;Gt(ROSA)26Sor^CreERT2^* embryos at E12.5 and cultured for 72h in the presence or absence of 4-hydroxytamoxifen (4-OHT). The phenotypes of the explants treated with 4-OHT (knockout explants) were compared to those of contralateral explants treated with a vehicle (ethanol) (control explants). The administration of 4-OHT to wild-type kidney explants did not cause any noticeable changes (data not shown). The knockout efficiency of *Bmp7* was approximately 90% in knockout explants (**Figure 3**). Jagged1 expressed in renal vesicles, comma-shaped bodies, and S-shaped bodies [19] was utilized to monitor the differentiation of cap mesenchyme after the epithelialization, whereas cytokeratin was utilized as a marker for branching ureteric buds. Jagged1-positive areas in *Bmp7* knockout explants significantly expanded compared to those in control kidneys (**Figure 3**), indicating the accelerated differentiation of cap mesenchyme in knockout explants. Although not significant, there was a tendency for the branching of ureteric buds to decrease in knockout kidneys (**Figure S4**).

### Bmp7 inhibits the epithelialization of cap mesenchyme in colony-forming assay

**Figure 4:**
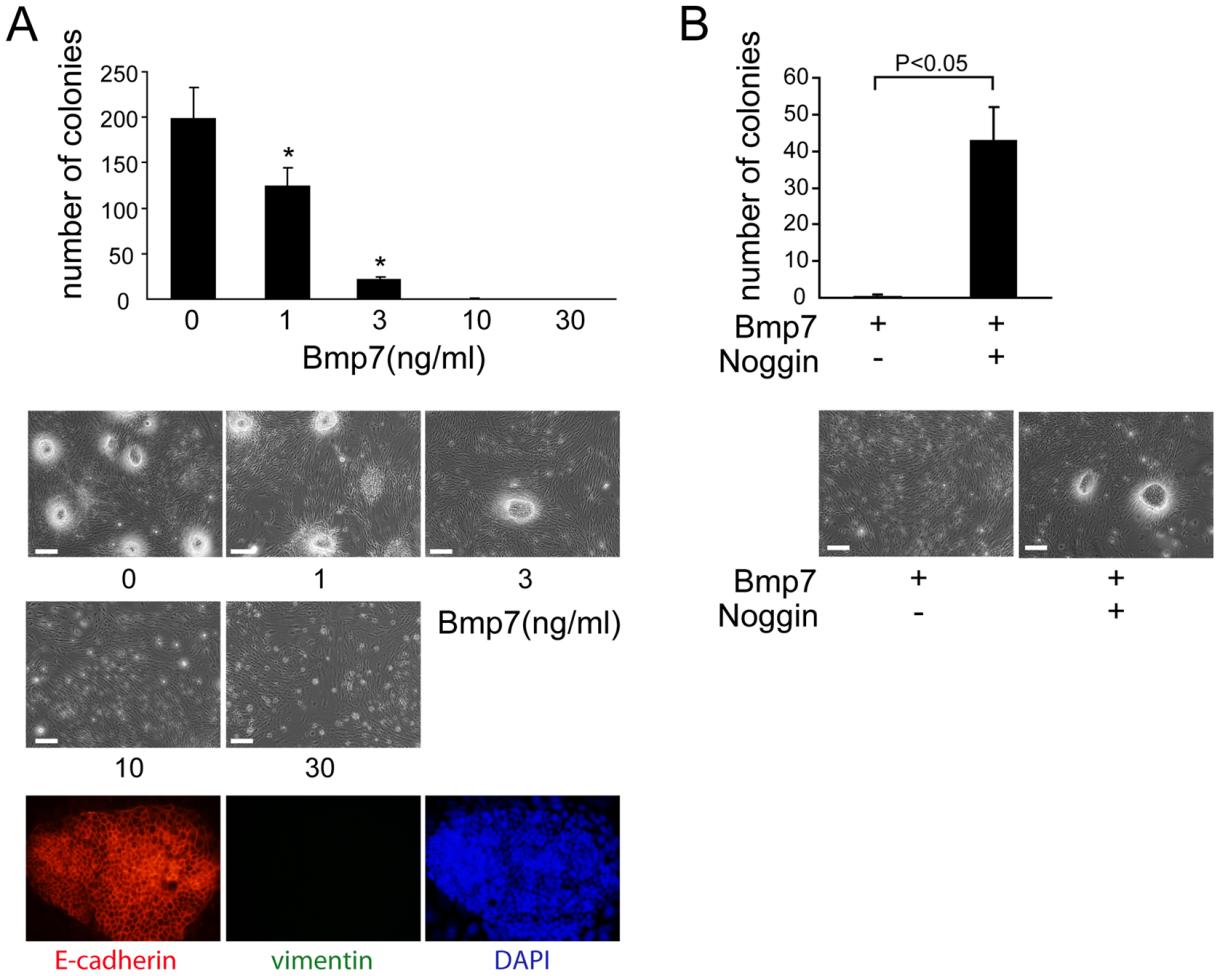
Bmp7 inhibits the epithelialization of cap mesenchyme in colony-forming assay. (**A**) Administration of Bmp7 dose-dependently inhibited the formation of sheetlike colonies of cap mesenchyme (n = 3). Data are represented as mean ± SD. Scale bars: 100 µm. The expression of E-cadherin, but not vimentin, in these colonies was confirmed by immunostaining. Because the colonies were formed on the feeder cells, some DAPI-positive nuclei were observed outside of the colony. (**B**) Simultaneous administration of Noggin (300 ng/ml) reversed the inhibitory effect of Bmp7 (10 ng/ml) on colony formation (n = 3). Data are represented as mean ± SD. *: P<0.001. Scale bars: 100 µm.

We also investigated the effect of Bmp7 on the epithelialization of cap mesenchyme using a colony-forming assay, in which the coculture of single cells from cap mesenchymes of E11.5 kidneys with Wnt4 expressing feeder cells (3T3Wnt4) [20] resulted in the epithelialization of cap mesenchyme and the formation of sheet-like E-cadherin-positive colonies (**Figure 4A**), as described previously [9]. This system is utilized as an assay to monitor the epithelialization of cap mesenchyme.

The addition of Bmp7 to this culture system significantly inhibited colony formation in a dose-dependent manner (**Figure 4A**). The simultaneous administration of Noggin, a Bmp antagonist, reversed the inhibitory effect of Bmp7 and rescued the epithelialization (**Figure 4B**). The prevalence of Ki67-positive cells in Bmp7-treated colonies was similar to that in vehicle-treated colonies (data not shown), indicating that the reduction of colony formation in the presence of Bmp7 was not due to attenuated cellular proliferation. These data strongly suggest that Bmp7 inhibits the differentiation of cap mesenchyme.

## Discussion

In this study, we demonstrate that Bmp7 inhibits the accelerated differentiation of kidney progenitor cells by utilizing three models, systemic inducible knockout mice, kidney explants and colony-forming assay. In systemic knockout mice, we observed the maturation of glomeruli at both E14.5 and E18.5, and of proximal tubules at E18.5, which supports the idea that Bmp7 inhibits the differentiation of progenitor cells. In kidney explants, we demonstrated the significant enlargement of Jagged1-positive nephron structures in knockout kidneys, which also indicates that Bmp7 inhibits the differentiation of progenitor cells. Finally we performed colony-forming assay to directly confirm that Bmp7 inhibits the epithelialization of progenitor cells.

The role of Bmp7 on the differentiation of progenitor population has been controversial. Vukicevic et al. reported that Bmp7 induces the differentiation of metanephric mesenchyme [21], whereas Dudley et al. [22] and Godin et al. [13] demonstrated opposing results showing that Bmp7 inhibits the differentiation of metanephric mesenchyme and more recently, Barak et al. demonstrated that Bmp7 synergizes with Fgf9 to promote survival and competence on nephron progenitor cells in vitro [23].

The controversy might be explained by the various effects of Bmp7 at different time points, and the various definitions of “differentiation”. In the paper of Vukicevic et al., the kidney explant treated with antisense oligonucleotides of Bmp7 was significantly thinner compared to controls, possibly due to the loss (apoptosis) of metanephric mesenchyme caused by the reduction of endogenous Bmp7, which is also supported by the reduction of Pax2. What we observed in conditional knockout mice, kidney explants and colony assay in this manuscript is the inhibitory effect of Bmp7 on the epithelialization of the induced metanephric mesenchyme, which is the next step after the induction of mesenchyme.

The phenotype of germline knockout mice is the massive apoptosis of renal progenitor cells, which precludes the analysis of Bmp7 function on progenitor cell differentiation [11], [12]. In our systemic knockout mice utilizing inducible Cre, the cessation of *Bmp7* expression at E12.5 permits the initial expansion of progenitor cells and enables us to observe the precocious differentiation of progenitor cells in the absence of Bmp7. This phenotype is novel, and could not be observed in germline *Bmp7* null mice due to massive apoptosis of metanephric mesenchyme.

Whether the differentiation in *Bmp7* knockout kidneys is “earlier” or “faster” remains unproven. However, the results of kidney explants and colony-forming assay showed Bmp7 inhibits epithelialization of mesenchyme, which is the early step of differentiation, and hence, at least, the differentiation in *Bmp7* knockout kidneys seems to occur “earlier”, while “faster” differentiation cannot be denied.

During tissue development, tissue-specific progenitor cells divide and populate the progenitor pool. The size of the progenitor pool as well as the final size of the tissues is determined by the balance between proliferation, apoptosis and differentiation of progenitor cells. Both massive apoptosis and precocious differentiation of progenitor cells causes depletion of the progenitor cells and reduces the final size of the tissues [24], [25], [26]. In this study, we discovered the novel function of Bmp7 to inhibit the precocious differentiation of progenitor cells. This novel function of Bmp7 together with its anti-apoptotic function [11], [12] coordinately maintains renal progenitor pool and determines the nephron number at birth.

Interestingly, the maintenance of stem/progenitor cells by Bmp may be an evolutionally conserved mechanism in different tissue contexts. It is widely accepted that orthologues of Bmps, Decapentaplegic (Dpp) and Glass bottom boat (Gbb) maintain germ line stem cells via the phosphorylation of Mad, a homologue of Smad proteins in drosophila ovary and testis [27], [28]. We also demonstrated that the inhibition of Smad signaling in kidney explant culture accelerated the differentiation of progenitor cells **(Figure S5)**, indicating the possible contribution of Bmp7-Smad signaling pathway in the maintenance of renal progenitors. More recently, the maintenance of germ line stem cells by Bmp7 has been demonstrated in mouse embryos as well [29]. In addition, it was reported that cerebellar neural progenitors was maintained by Bmp7 secreted from the choroid plexus [30].

There are several possible sources of Bmp7 that contribute to the phenotype in *Bmp7* knockout mice: maternal, extrarenal, and intrarenal Bmp7. Previous reports demonstrated that maternal Bmp7 traverses the placental barrier and localizes in embryonic kidneys until day 14 of gestation [31]. In our analysis, however, the genotypes of pregnant mice are designed to be *Bmp7^+/LacZ^;Gt(ROSA)26Sor^CreERT2^*or *Bmp7^fl/fl^*, such that the amount of Bmp7 originating from the mother is unaltered by the administration of tamoxifen, excluding the possibility that the fluctuation of maternal Bmp7 levels contributes to the phenotypes in *Bmp7* knockout embryos. Given the nature of systemic inducible *Gt(ROSA)26Sor^CreERT2^*mice, the expression of *Bmp7* in other tissues was also reduced after tamoxifen treatment, so that the circulating level of Bmp7 (if present) should be lowered. This might contribute to the phenotypes in *Bmp7* knockout embryos to some extent. However, the accelerated differentiation of cap mesenchyme was observed in kidney explant culture, in which the ablation of *Bmp7* occurs intrinsically within the kidney, indicating that intrarenal Bmp7 is primarily responsible for the phenotypes.

Interestingly, the inactivation of *Bmp7* in distinct cell types manifests different spectrums of defects in the kidney. Thus, the loss of Bmp7 from podocytes results in the reduction of proximal tubules in postnatal kidneys [18], whereas systemic *Bmp7* knockout mice (this study) exhibits the accelerated maturation of nephrons. These comparisons indicate that there is a broad range of functions for Bmp7 in different cell types at different stages, and raise the possibility that Bmp7 secreted from one cell population functions locally in an autocrine or paracrine fashion to exert a unique function. This hypothesis is strengthened by a previous report indicating that the prodomain of Bmp7 tethers Bmp7 to the extracellular matrix (ECM) near the site of Bmp7 production and inhibits its passive diffusion [32].

Finally, our analysis demonstrates for the first time that Bmp7 plays a critical role in the maintenance of the renal progenitor population in the developing kidney, and thereby determines the final number of nephrons. Further analysis will elucidate the mechanism how intrauterine conditions affect the pathway, and provide a rationale for a therapeutic approach to prevent congenital deficit in nephron number.

## Methods

### Ethics Statement

All animal studies were approved by the Animal Research Committee, Graduate School of Medicine, Kyoto University (permission number: MedKyo 12522), and were strictly in accordance with the Guide for the Care and Use of Laboratory Animals of the National Institutes of Health. Mice were sacrificed by cervical dislocation by well-trained researchers.

### Animal use

The *Bmp7* conditional allele [14] and *Gt(ROSA)26Sor^CreERT2^* allele were generated at Regeneron Pharmaceuticals using Velocigene™ technology as described elsewhere [33]. The *Bmp7^+/LacZ^* knock-in allele has been described previously [11], [13]. We bred *Bmp7^+/LacZ^;Gt(ROSA)26Sor^CreERT2^* mice with *Bmp7^fl/fl^* mice to yield *Bmp7^LacZ/fl^;Gt(ROSA)26Sor^CreERT2^* (*Bmp7* knockout) and *Bmp7^+/fl^;Gt(ROSA)26Sor^CreERT2^* (control) embryos. Due to the hematological toxicity of *Gt(ROSA)26Sor^CreERT2^* mice [15], all experiments were planned for both knockout and control embryos to have one *CreERT2* allele.

### Administration of tamoxifen

Tamoxifen (Sigma, St. Louis, MO, USA) was dissolved in a sunflower oil/ethanol (9:1) mixture at 30 mg/ml. For activation of CreERT2, the tamoxifen solution was administered at a concentration of 150 mg/kg by intraperitoneal injection to pregnant mothers bearing *Bmp7^LacZ/fl^;Gt(ROSA)26Sor^CreERT2^* and *Bmp7^+/fl^;Gt(ROSA)26Sor^CreERT2^* embryos.

### Renal histopathology

The kidneys were fixed in Carnoy’s Solution, and embedded in paraffin. Sections (2 µm thick) were sliced at every 100 µm (the diameter of any glomeruli was shorter than 100 µm) and stained with periodic acid-Schiff (PAS) for routine histological examination. Frozen sections of the kidneys were immunostained as previously described [34], [35]. The primary antibodies were against WT1 (Santa Cruz Biotechnology Inc., Santa Cruz, CA, USA), phospho-Histone H3 (Upstate, Lake placid, NY, USA), nephrin (PROGEN, Germany), THP (Biomedical Technologies, Stoughton, MA, USA), pan-cytokeratin (Sigma, BD Transduction Laboratories, Franklin Lakes, NJ, USA), Jagged1 (Santa Cruz Biotechnology Inc.), E-cadherin (Sigma), and vimentin (Cell Signaling Technology, Danvers, MA, USA). Alexa-conjugated secondary antibodies were purchased from Molecular Probes (Eugene, OR, USA). FITC-conjugated lotus lectin (LTA) (Seikagaku Kogyo, Japan) was used to visualize proximal tubules. WT1-positive area was measured utilizing Photoshop software. Terminal deoxynucleotidyl transferase dUTP nick end labeling (TUNEL) staining was performed with an In Situ Apoptosis Detection Kit (Takara Bio Inc., Japan), and nuclei were counterstained with methyl green.

### Quantification of maturation of glomeruli

Maturation of glomeruli was analyzed in five (E14.5) or three (E18.5) serial sections (including the section with renal hylus) at every 100 µm of the embryonic kidneys for 7 control embryos and 5 knockout embryos at E14.5, and 4 control embryos and 6 knockout embryos at E18.5.

The criteria for the assessment of glomerular maturation are based on the previous publications [16], [17]. C stage: Cells of the lower limb of the S-shaped body differentiate to form an immature, crescent-shaped glomerulus. Stage I: Glomeruli with at least half of the glomerular tuft lined with dark-staining cuboidal podocytes. Stage II: Glomeruli with less than half the circumference of the tuft lined with dark cuboidal podocytes, with at least five adjoining. Stage III: Glomeruli with typical flattened podocytes.

We counted the number of glomeruli in each stage in the serial sections and showed the sum number in Figure 1 and 2.

### Quantification of proximal tubule complexity

Scoring of proximal tubule cross-sections was performed according to the previous publication [18]. Briefly, we stained three frozen sections in each embryo and counted the number of LTA-positive proximal tubule cross-sections as well as the number of WT1-positive glomeruli in each section. The number of proximal tubule cross-sections per glomeruli is considered to represent the complexities of proximal tubules, which should correlate with the length of proximal tubules.

### Quantification of mRNA by real-time reverse transcription polymerase chain reaction

Real-time reverse transcription (RT) polymerase chain reaction (PCR) was performed as described previously [36], [37]. A 7300 Fast Real-Time PCR system (Applied Biosystems, Fostercity, CA, USA) was used. Specific primers were designed using Primer Express software (Applied Biosystems). The sequences of the primers used were as follows:

*GAPDH*, CCAGAACATCATCCCTGCATC; CCTGCTTCACCACCTTCTTGA,

*Bmp7*, TGTGGCAGAAAACAGCAGCA; TCAGGTGCAATGATCCAGTCC

Serially diluted cDNA was used to generate the standard curve for each primer, and the PCR conditions were as follows: 50°C for 2 min, 95°C for 10 min, then 95°C for 15 sec, and 60°C for 1 min for 40 cycles. The results were normalized with the amount of *GAPDH* gene.

### Kidney explant culture

Kidney explant culture was performed as previously described [38]. Briefly, fresh kidney explants were isolated from mouse embryos and cultured on polycarbonate filters (0.4 µm pore size, Nucleopore) floating on DMEM10%FCS at 37°C in 5% CO_2_.

To knockout *Bmp7 in vitro*, one side of E12.5 embryonic kidneys from *Bmp7^LacZ/fl^;Gt(ROSA)26Sor^CreERT2^* embryos was treated with 1 µM 4-OHT (Sigma) dissolved in ethanol, while contralateral kidney was treated with vehicle (ethanol), and cultured for 72 h. Neither *Bmp7^LacZ/fl^;Gt(ROSA)26Sor^CreERT2^* explants treated with the vehicle or *Bmp7^+/fl^;Gt(ROSA)26Sor^CreERT2^* explants treated with 4-OHT exhibited any morphological changes. To cancel the effect of individual difference between embryos, the results of *Bmp7^LacZ/fl^;Gt(ROSA)26Sor^CreERT2^*kidneys treated with the vehicle were shown as controls in comparison with contralateral kidneys from the same embryos treated with 4-OHT. For immunostaining, cultured kidney explants were fixed in 4% paraformaldehyde, quenched with 50 mM NH_4_Cl, permeabilized by 0.1% triton X, and stained as previously described [38]. Jagged1-positive area was measured utilizing Photoshop software.

To inhibit Smad signaling in kidney explants, one side of E12.5 embryonic kidneys from wild-type mice were treated with 0.5 µM dorsomorphin (Calbiochem) dissolved in DMSO, while contralateral kidney was treated with vehicle (DMSO), and cultured for 48 h.

### Immunoblotting

Immunoblotting was performed as described previously [35]. Cultured kidney explants were suspended in RIPA buffer for protein extraction. The primary antibodies were anti-phospho-Smad1/5/8 (Cell Signaling Technology), and anti-GAPDH (Fitzgerald Industries).

### Colony-forming assay

A colony-forming assay was performed as described previously [9]. Single cap mesenchymal cells from E11.5 ICR mice were plated onto feeder cells expressing Wnt4 (a kind gift from Professor Kispert) [20] at a density of 1.5×10^3^ cells/well in 12-well plates and treated with recombinant Bmp7 (R&D systems Inc., Minneapolis, MN, USA, Pro Spec, Israel) with or without recombinant Noggin-Fc chimera (R&D systems Inc.) 24 h later. After 7 days of culture, the number of colonies was counted.

### Statistical analysis

All assays were performed at least three times. Data are presented as the mean ± standard deviation (SD). Statistical significance was assessed by Student’s *t*-test for two group comparisons, and the ANOVA for more than three.

## Supporting Information

Figure S1 **Systemic knockout of Bmp7 at E10.5 recapitulates the phenotypes of germline Bmp7 knockout mice.** Pregnant mothers bearing both Bmp7^+/fl^;Gt(ROSA)26Sor^CreERT2^ and Bmp7^LacZ/fl^;Gt(ROSA)26Sor^CreERT2^ embryos were administered tamoxifen at E10.5, and sacrificed at E16.5. The Bmp7^LacZ/fl^;Gt(ROSA)26Sor^CreERT2^ (Bmp7 knockout) kidney was smaller and exhibited severe reduction of cap mesenchyme. Scale bars: 100 µm.
(TIF)

Figure S2 **Volume of kidneys and mesenchyme is still maintained in Bmp7 knockout embryos at E14.5, but reduces at E18.5 (related to** **Figure 1C-E** **and** **2C-F** **).** The maximum horizontal sectional area of kidneys and the thickness of nephrogenic zone, WT1-positive area were not different between Bmp7 knockout embryos (white column) and controls (black column) at E14.5, but significantly reduced in knockout embryos at E18.5. The mean of the values ± SD is presented in the graphs (At E14.5, n = 7 for control embryos, and 5 for knockout embryos. Thickness of nephrogenic zone and WT1-positive area were measured in 5 slices of each kidney. At E18.5, n = 4 for control embryos, and 6 for knockout embryos. Thickness of nephrogenic zone and WT1-positive area were measured in 3 slices of each kidney.) n.s.: not significant.
(TIF)

Figure S3 **Accerelated maturation of distal tubules in Bmp7 knockout kidneys at E18.5 (related to** **Figure 2L, M** **).** (A) Kidneys were stained with nephrin (green) and THP (red) to label glomeruli and distaltubules, respectively. The volume of THP-positive distal tubule sections in Bmp7 knockout kidneys was comparable to the control kidneys, whereas the number of glomeruli was significantly reduced. (B) The number of THP+ distal tubule cross sections normalized by the number of nephrin+ glomeruli tended to increase in knockout kidneys (white column) compared to control kidneys (black column). Data are represented as mean ± SD. Three sections were stained for each kidney. The sum of the number of THP-positive distal tubule cross sections was divided by the sum of the number of nephrin-positive glomeruli. The mean of the values from five (control) or six (knockout) embryos is presented in the graph. Scale bars: 100 µm. n.s.: not significant.
(TIF)

Figure S4 **The branching of ureteric buds tends to decrease in Bmp7 knockout kidneys (related to** **Figure 3** **).** Kidney explants were taken from Bmp7^+/fl^;Gt(ROSA)26Sor^CreERT2^ and Bmp7^LacZ/fl^;Gt(ROSA)26Sor^CreERT2^ embryos at E11.5 and cultured for 48 h with or without 4-OHT. In Bmp7^+/fl^;Gt(ROSA)26Sor^CreERT2^ embryos, the number of ureteric bud tips of tamoxifen-treated explants was almost equal to vehicle-treated explants. In Bmp7^LacZ/fl^;Gt(ROSA)26Sor^CreERT2^ embryos, although the difference was not significant, the number of ureteric bud tips of tamoxifen-treated (Bmp7 knockout) explants tended to decrease compared to vehicle-treated explants. Data are represented as mean ± SD (n = 4). n.s.: not significant.
(TIF)

Figure S5 **Smad signaling inhibits the differentiation of cap mesenchyme in the kidney explant culture (related to** **Figure 3** **).** Kidney explants were taken from wild-type mice at E12.5 and cultured for 48 h in the presence or absence of a Smad1/5/8 inhibitor, dorsomorphin. In explants treated with dorsomorphin, Jagged1-positive regions were significantly expanded. Scale bars: 100 µm. Data are represented as mean ± SD (n = 8). Immunoblotting of the lysates of kidney explants demonstrated the phosphorylation of Smad1/5/8 was decreased in dorsomorphin-treated explants. Ten micrograms of kidney explants lysate was loaded in each lane. As a positive control, primary kidney cells were stimulated with 100 ng/ml Bmp7 for 1 h. GAPDH was used as a loading control. pSmad1/5/8 denotes phospho-Smad1/5/8.
(TIF)
