# Supplementary figures and images for: Bmp7 Maintains Undifferentiated Kidney Progenitor Population and Determines Nephron Numbers at Birth
Source: PLoS One. 2013 Aug 26;8(8):e73554. doi: 10.1371/journal.pone.0073554 (PMC3753328; doi:10.1371/journal.pone.0073554)

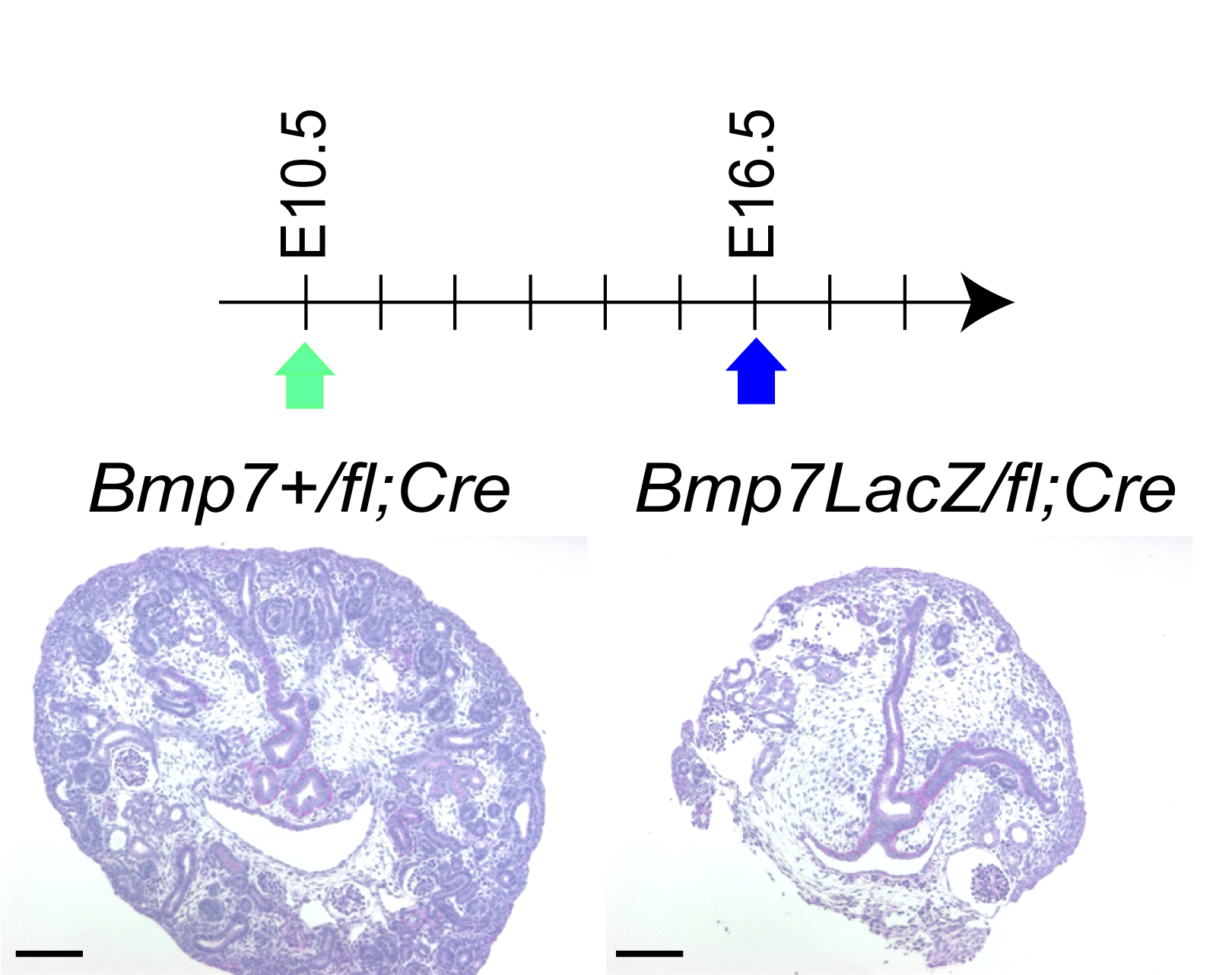

Supplement: Figure S1 — Systemic knockout of Bmp7 at E10.5 recapitulates the phenotypes of germline Bmp7 knockout mice. Pregnant mothers bearing both Bmp7+/fl;Gt(ROSA)26SorCreERT2 and Bmp7LacZ/fl;Gt(ROSA)26SorCreERT2 embryos were administered tamoxifen at E10.5, and sacrificed at E16.5. The Bmp7LacZ/fl;Gt(ROSA)26SorCreERT2 (Bmp7 knockout) kidney was smaller and exhibited severe reduction of cap mesenchyme. Scale bars: 100 µm. (TIF) [file pone.0073554.s001.tif]

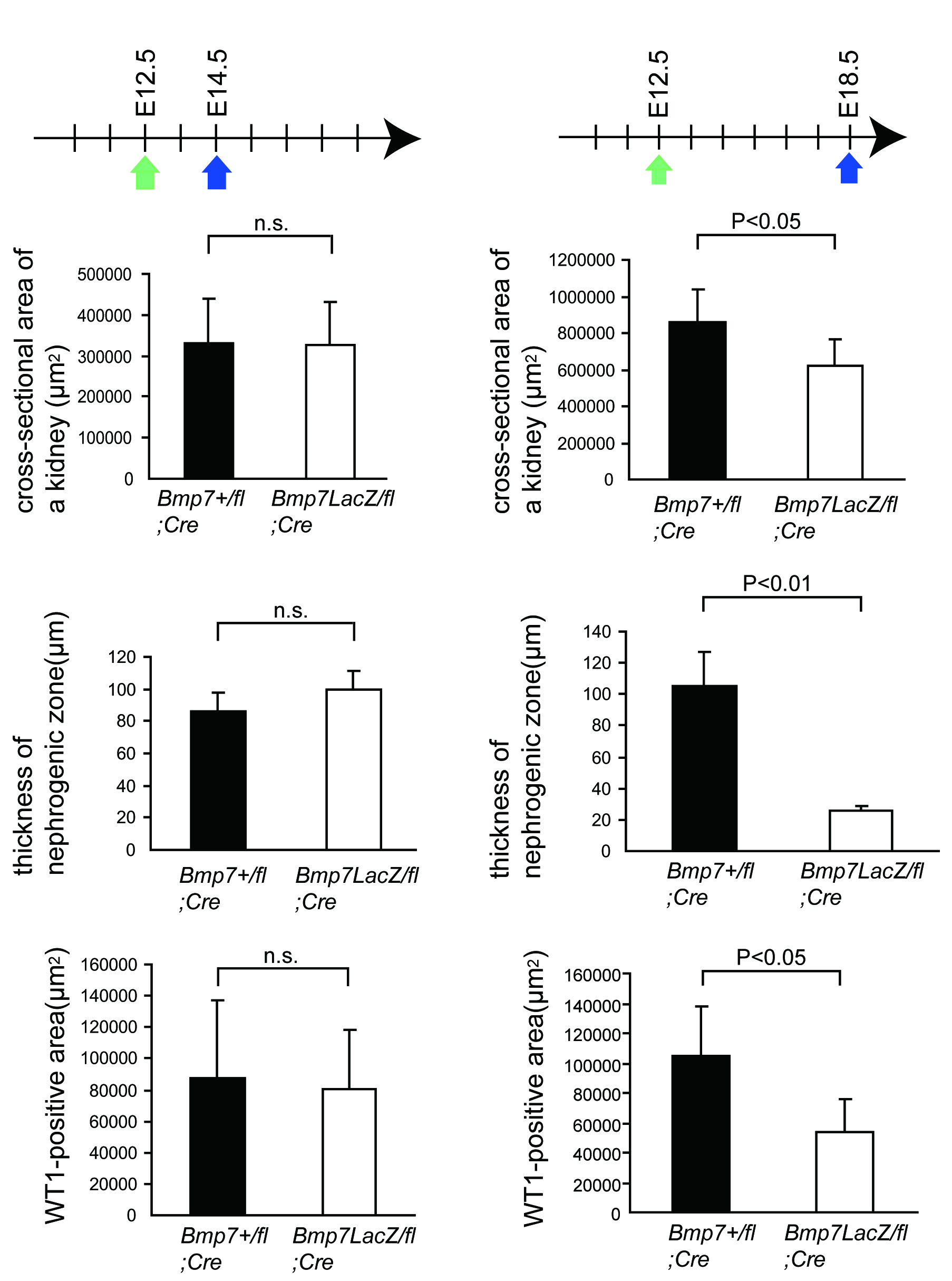

Supplement: Figure S2 — Volume of kidneys and mesenchyme is still maintained in Bmp7 knockout embryos at E14.5, but reduces at E18.5 (related to Figure 1C-E and 2C-F ). The maximum horizontal sectional area of kidneys and the thickness of nephrogenic zone, WT1-positive area were not different between Bmp7 knockout embryos (white column) and controls (black column) at E14.5, but significantly reduced in knockout embryos at E18.5. The mean of the values ± SD is presented in the graphs (At E14.5, n = 7 for control embryos, and 5 for knockout embryos. Thickness of nephrogenic zone and WT1-positive area were measured in 5 slices of each kidney. At E18.5, n = 4 for control embryos, and 6 for knockout embryos. Thickness of nephrogenic zone and WT1-positive area were measured in 3 slices of each kidney.) n.s.: not significant. (TIF) [file pone.0073554.s002.tif]

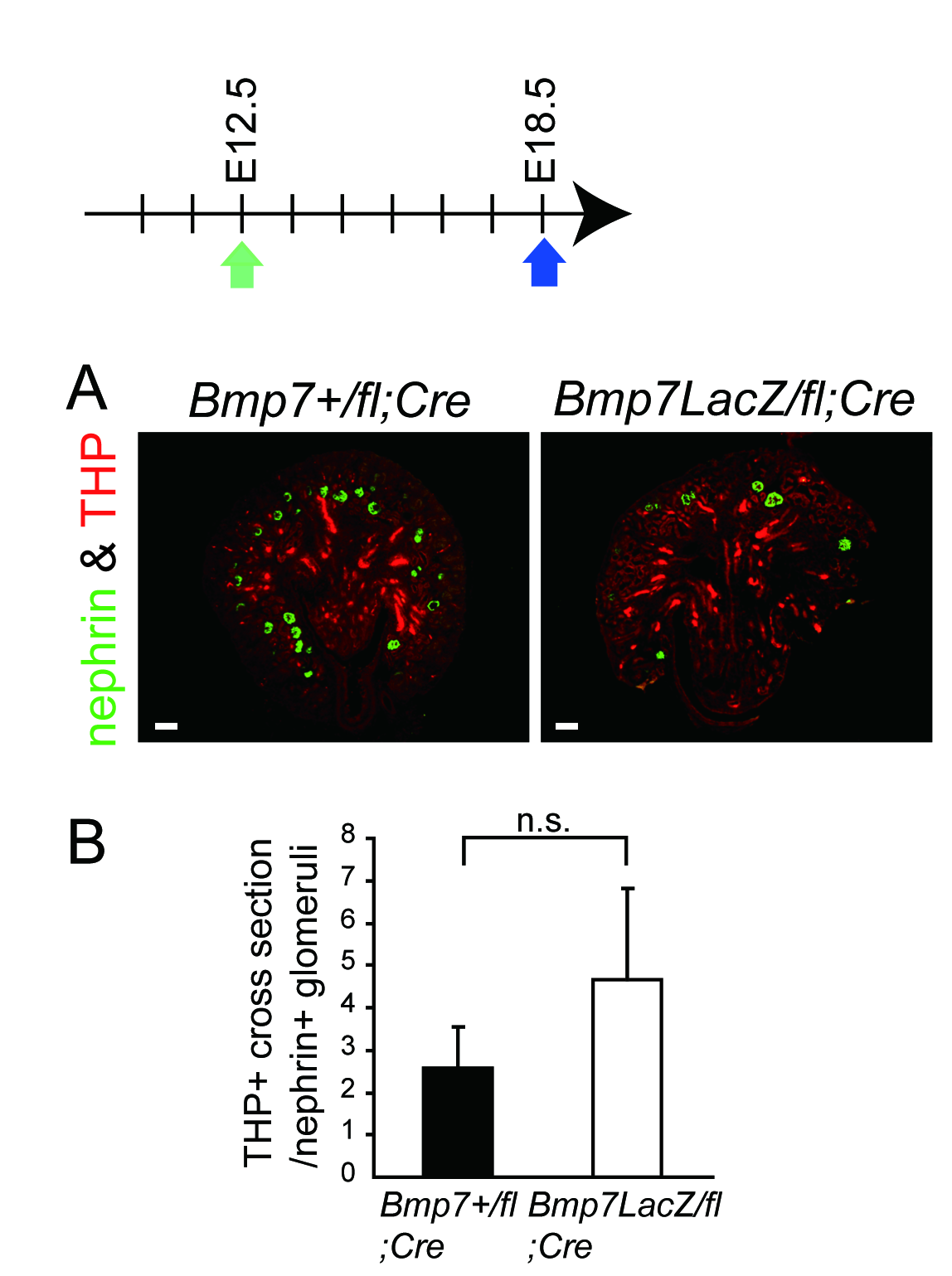

Supplement: Figure S3 — Accerelated maturation of distal tubules in Bmp7 knockout kidneys at E18.5 (related to Figure 2L, M ). (A) Kidneys were stained with nephrin (green) and THP (red) to label glomeruli and distaltubules, respectively. The volume of THP-positive distal tubule sections in Bmp7 knockout kidneys was comparable to the control kidneys, whereas the number of glomeruli was significantly reduced. (B) The number of THP+ distal tubule cross sections normalized by the number of nephrin+ glomeruli tended to increase in knockout kidneys (white column) compared to control kidneys (black column). Data are represented as mean ± SD. Three sections were stained for each kidney. The sum of the number of THP-positive distal tubule cross sections was divided by the sum of the number of nephrin-positive glomeruli. The mean of the values from five (control) or six (knockout) embryos is presented in the graph. Scale bars: 100 µm. n.s.: not significant. (TIF) [file pone.0073554.s003.tif]

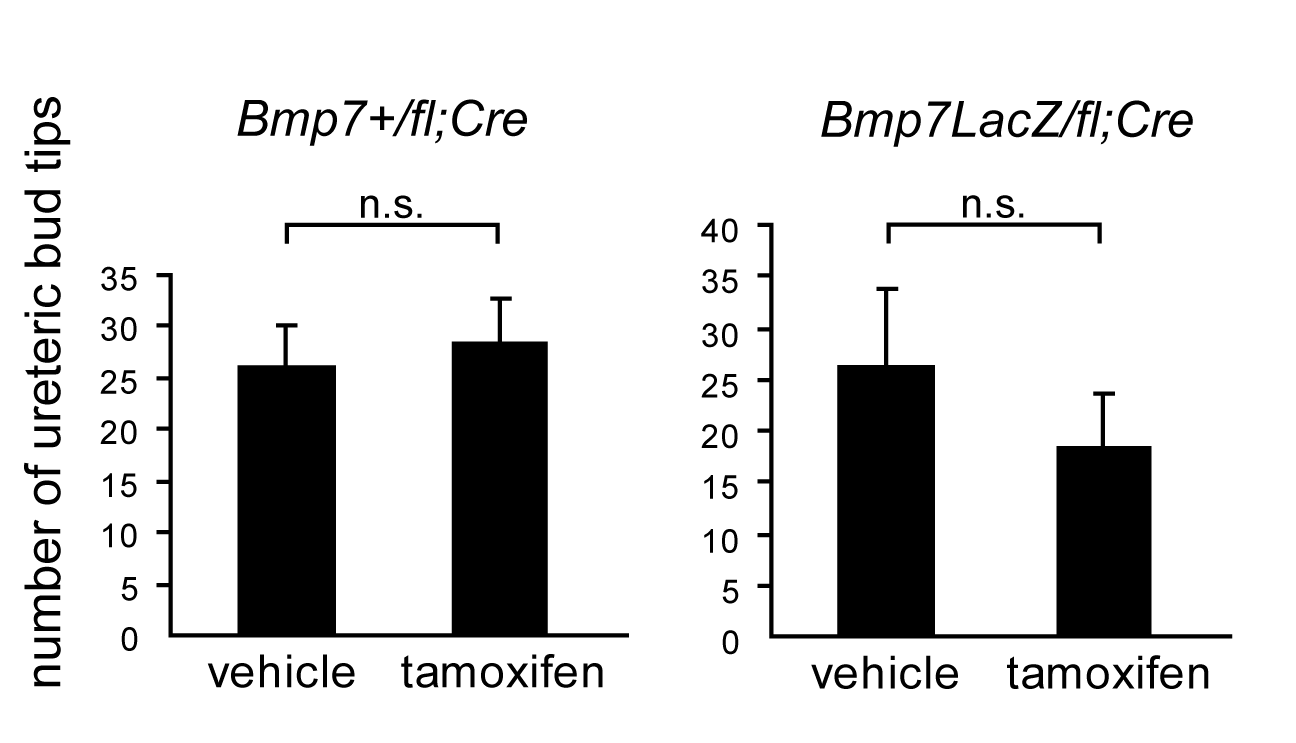

Supplement: Figure S4 — The branching of ureteric buds tends to decrease in Bmp7 knockout kidneys (related to Figure 3 ). Kidney explants were taken from Bmp7+/fl;Gt(ROSA)26SorCreERT2 and Bmp7LacZ/fl;Gt(ROSA)26SorCreERT2 embryos at E11.5 and cultured for 48 h with or without 4-OHT. In Bmp7+/fl;Gt(ROSA)26SorCreERT2 embryos, the number of ureteric bud tips of tamoxifen-treated explants was almost equal to vehicle-treated explants. In Bmp7LacZ/fl;Gt(ROSA)26SorCreERT2 embryos, although the difference was not significant, the number of ureteric bud tips of tamoxifen-treated (Bmp7 knockout) explants tended to decrease compared to vehicle-treated explants. Data are represented as mean ± SD (n = 4). n.s.: not significant. (TIF) [file pone.0073554.s004.tif]

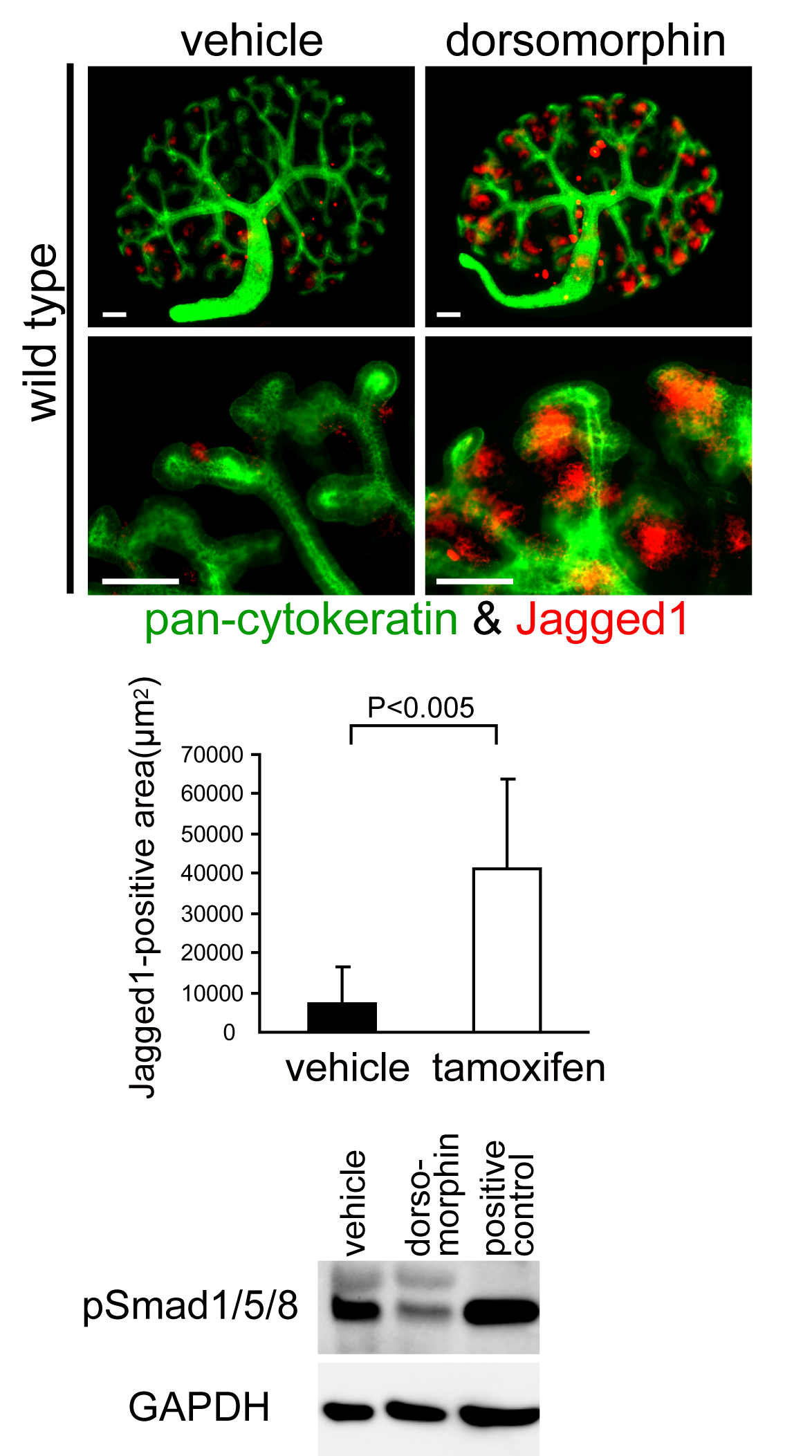

Supplement: Figure S5 — Smad signaling inhibits the differentiation of cap mesenchyme in the kidney explant culture (related to Figure 3 ). Kidney explants were taken from wild-type mice at E12.5 and cultured for 48 h in the presence or absence of a Smad1/5/8 inhibitor, dorsomorphin. In explants treated with dorsomorphin, Jagged1-positive regions were significantly expanded. Scale bars: 100 µm. Data are represented as mean ± SD (n = 8). Immunoblotting of the lysates of kidney explants demonstrated the phosphorylation of Smad1/5/8 was decreased in dorsomorphin-treated explants. Ten micrograms of kidney explants lysate was loaded in each lane. As a positive control, primary kidney cells were stimulated with 100 ng/ml Bmp7 for 1 h. GAPDH was used as a loading control. pSmad1/5/8 denotes phospho-Smad1/5/8. (TIF) [file pone.0073554.s005.tif]
